# Genome Sequences of 28 *Bordetella pertussis* U.S. Outbreak Strains Dating from 2010 to 2012

**DOI:** 10.1128/genomeA.01075-13

**Published:** 2013-12-19

**Authors:** Eric T. Harvill, Laura L. Goodfield, Yury Ivanov, Jessica A. Meyer, Christopher Newth, Pamela Cassiday, Maria Lucia Tondella, Patty Liao, Jerry Zimmerman, Kathleen Meert, David Wessel, John Berger, J. Michael Dean, Richard Holubkov, Jeri Burr, Teresa Liu, Lauren Brinkac, Maria Kim, Liliana Losada

**Affiliations:** The Pennsylvania State University, University Park, Pennsylvania, USAa; NICHD Collaborative Pediatric Critical Care Research Network, Salt Lake City, Utah, USAb; Centers for Disease Control and Prevention, Atlanta, Georgia, USAc; University of Utah Data Coordinating Center, Salt Lake City, Utah, USAd; J. Craig Venter Institute, Rockville, Maryland, USAe

## Abstract

Despite the availability of highly effective vaccines, *Bordetella pertussis* incidence has been rapidly rising in highly vaccinated populations. Recent outbreaks have received media attention, feeding concerns about the emergence of dangerous new strains with increased virulence or that escape vaccine-induced immunity. To accelerate the study of this reemerging pathogen, we sequenced the genomes of 28 *B. pertussis* strains isolated during outbreaks from 2010 through 2012, making both strains and sequence data available to the scientific community.

## GENOME ANNOUNCEMENT

The Centers for Disease Control and Prevention classifies whooping cough as a reemerging disease, documenting increasing numbers of cases nearly every year, from hundreds per year in the 1970s to >41,000 in 2012 (1). The recent high-profile epidemics in California (in 2010) and Washington (in 2012) contribute to growing concerns and feed speculation about the ongoing evolution of *Bordetella pertussis*. To address these concerns, we collected 28 strains of *B. pertussis* from these and other outbreaks and made them available at the Biodefense and Emerging Infections Research Resources Repository for further study by others.

Here, we report the genome sequences of these 28 clinical isolates derived from whooping cough hospital cases that occurred between 2010 and 2012. Genomic DNA was prepared using a phenol-chloroform extraction method and ethanol precipitation (2). A combination of 3- or 5-kb mate pair (~30× coverage) and 100-bp Illumina paired-end reads (~50× coverage) were used for genome sequence determination. After quality trimming, all reads were used to generate assemblies with Celera assembler 6.1 (3) or Velvet assembler (4). After improvement, all genomes had between 4 and 186 scaffolds containing 25 to 285 contigs. Underlying consensus sequences and gaps were improved using custom scripts developed at the J. Craig Venter Institute (JCVI). The overall G+C content in all cases was ~67%, with genome sizes ranging from 3.83 Mb to 4.15 Mb (average, 4.0 Mb). Up to 218 copies of insertion element IS*481* and 95 tripartite transcarboxylate transporter (TTT) element were found in any one *B. pertussis* genome, amounting to >215 kb on average. All of the *B. pertussis* isolates, irrespective of geographic location, belong to the same multilocus sequence type and are nearly identical to each other at the protein level (average 99% identity) in conserved regions of the chromosome. However, the *B. pertussis* genomes were subject to massive genome rearrangements and different gene losses that account for the majority of the diversity between strains. All the genomes were annotated using JCVI’s annotation pipeline (http://www.jcvi.org) and were predicted to have between 3,750 and 4,193 genes. As expected, mobile elements were overrepresented in these genomes compared to in published *Bordetella bronchiseptica* genomes due to the expansion of the repetitive elements. Over 1,000 core *B. bronchiseptica* genes were absent in all of the *B. pertussis* genomes. Many of these genes are involved in capsule biosynthesis, alternate respiration, nutrient acquisition, type VI secretion, and antibiotic resistance. All *B. pertussis* isolates encoded pertussis toxin, filamentous hemagglutinin, type III secretion system, adenylate cyclase, and other virulence factors.

The findings from this study suggest that currently circulating *B. pertussis* isolates in the United States are derived from a single genetic background. A full analysis of the virulence genes and evolution of *B. pertussis* is under way and will be published in a subsequent report.

### Nucleotide sequence accession numbers.

The *B. pertussis* whole-genome shotgun projects have been deposited for each isolate at DDBJ/EMBL/GenBank as described in Table 1. The version described in this paper is the first version.

**Table 1:** Characteristics of the 28 *B. pertussis* strains

*B. pertussis* strain name	NCBI accession no.	No. of contigs	N_50_ (bp)	Total length (bp)	G+C %
2250905	AXSU00000000	285	20,908	3,940,180	67.76
2356847	AXST00000000	179	31,198	3,907,019	67.86
2371640	AXSS00000000	193	33,462	3,904,605	67.88
STO1-SEAT-0006	AXSR00000000	49	139,362	4,028,421	67.77
STO1-SEAT-0007	AXSQ00000000	26	343,155	4,053,851	67.78
STO1-CHLA-0011	AXSP00000000	25	419,274	4,083,349	67.69
H897	AXSO00000000	173	36,602	3,938,675	67.81
H918	AXSN00000000	60	150,397	4,073,712	67.71
H921	AXSM00000000	48	164,785	4,064,086	67.73
H939	AXSL00000000	43	177,196	4,018,810	67.74
H973	AXSK00000000	62	112,367	4,014,250	67.81
STO1-SEAT-0004	AXSJ00000000	121	52,170	3,839,596	67.9
I002	AXSI00000000	63	102,522	4,047,567	67.74
I036	AXSH00000000	46	189,616	4,057,285	67.74
I176	AXSG00000000	33	266,526	4,045,246	67.78
STO1-CHLA-0006	AXSF00000000	65	134,387	4,044,283	67.79
CHLA-15	AXSD00000000	138	46,158	3,844,206	67.89
CHLA-13	AXSE00000000	95	74,009	4,029,822	67.79
CHLA-20	AXSC00000000	131	46,140	3,828,499	67.9
CHLA-26	AXSB00000000	41	264,509	4,045,408	67.8
STO1-CHOC-0016	AXSA00000000	54	122,855	4,033,849	67.79
STO1-CHOC-0017	AXRZ00000000	108	45,197	4,050,208	67.8
STO1-CHOC-0018	AXRY00000000	127	43,391	4,051,577	67.81
STO1-CHOC-0019	AXRX00000000	120	52,255	4,150,262	67.72
STO1-CHOC-0021	AXRW00000000	56	115,723	4,027,480	67.8
STO1-CHOC-0008	AXRV00000000	124	50,507	3,834,602	67.9
STO1-CHOM-0012	AXRU00000000	37	222,972	4,065,443	67.71
STO1-CNMC-0004	AXSV00000000	149	46,159	3,855,042	67.88
